# Langerhans Cell Histiocytosis: A Diagnostic Challenge in the Oral Cavity

**DOI:** 10.1155/2017/1691403

**Published:** 2017-10-24

**Authors:** Mehmet Ali Altay, Alper Sindel, Öznur Özalp, Burak Kocabalkan, İrem Hicran Özbudak, Ramazan Erdem, Ozan Salim, Dale A. Baur

**Affiliations:** ^1^Department of Oral and Maxillofacial Surgery, Akdeniz University, Faculty of Dentistry, Antalya, Turkey; ^2^Department of Pathology, Akdeniz University, Faculty of Medicine, Antalya, Turkey; ^3^Department of Hematology, Hatay State Hospital, Hatay, Turkey; ^4^Department of Hematology, Akdeniz University, Faculty of Medicine, Antalya, Turkey; ^5^Department of Oral and Maxillofacial Surgery, Case Western Reserve University, School of Dental Medicine, Cleveland, OH, USA

## Abstract

**Background:**

Langerhans cell histiocytosis (LCH) is a rare disorder of the reticuloendothelial system with unknown etiology. This report aims to present a case of LCH with diffuse involvement of the oral cavity and to raise awareness of the distinguishing features of this diagnostically challenging entity.

**Case Report:**

A 26-year-old male patient presented with complaints of teeth mobility, intense pain, and difficulty in chewing. Intraoral and radiological examinations revealed generalized gingival hyperplasia and severe teeth mobility with widespread alveolar bone loss. Periodontal therapy was performed with no significant improvement. An incisional biopsy revealed Langerhans cells and positive reaction to S-100 and CD1, and the patient was diagnosed with LCH. The patient underwent systemic chemotherapy with vinca alkaloids and corticosteroids. Regression of gingival lesions, as well as significant decrease in mobility of the remaining teeth and severity of pain, was achieved during 12 months of follow-up.

**Conclusion:**

The rarity and variable system involvement of LCH necessitate a multidisciplinary approach be carried out for accurate diagnosis, effective treatment, and an uneventful follow-up. Awareness of oral manifestations of LCH may aid clinicians greatly in reducing morbidity and mortality associated with this debilitating condition.

## 1. Background

Previously termed “histiocytosis X,” Langerhans cell histiocytosis (LCH) is a rare disorder characterized by intense and abnormal proliferation of bone marrow-derived immature myeloid dendritic cells-Langerhans cells (LCs) in the skin, bone, lymph nodes, and other organs [1]. LCH more commonly occurs in children with a male predilection. The incidence of the disease is reported to be 8.9 per million in children and 1-2 cases per million in adult population [2, 3]. Birbeck granules and positive immunohistochemistry for S100 and CD1a are recognized as the standard diagnostic histological features of LCH. Despite its uniform histologic nature, LCH includes a broad spectrum of clinical manifestations that can vary from a self-limiting single bone disease to an aggressive fatal disseminated form [4].

Previously classified into three different clinical entities including eosinophilic granuloma, Hand–Schüller–Christian disease, and Letterer–Siwe disease, the current classification of LCH is made according to dissemination of the disease: single-system and multisystem LCH [2, 5]. Single-system LCH most commonly involves the bone followed by skin, lymph nodes, and the lung. Bone lesions generally occur in unifocal form and mostly affect skull, ribs, pelvic bones, long bones, vertebrae, and feet [6, 7].

LCH involves the head and neck region quite commonly and in particular the bones of the skull and jaws [8]. Gingiva and hard palate are the most commonly affected sites in maxillomandibular involvement [9]. Oral manifestations of LCH comprise ulceroproliferative and bleeding gingiva, mobile teeth, and pain which is reported to be the most common symptom [10]. The symptoms of LCH may occur first in oral cavity before elsewhere in the body. Therefore, a thorough examination as well as establishing and ruling out differential diagnoses has significant importance in reaching the diagnosis of LCH at an earlier stage [11].

The aim of this report is to present a case of LCH with generalized involvement of the oral cavity. Seeking to raise awareness of the distinguishing features of this diagnostically challenging entity among clinicians, clinical and histological features of the disease as well as current treatment options are discussed in detail.

## 2. Case Report

**Figure 1:**
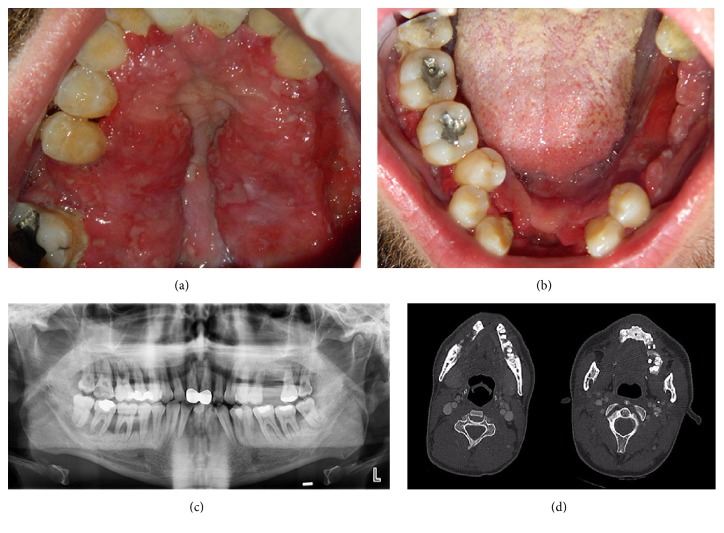
(a) Intraoral view of the patient after extraction of teeth with severe mobility, prior to initiation of chemotherapy. Extensive gingival lesions are observed on the palatal surface of the maxilla. (b) Intraoral view of the patient after extraction of teeth with severe mobility, prior to initiation of chemotherapy. Diffuse, erythematous, and ulcerated gingival hyperplasia is observed in the mandible. (c) Panoramic radiograph of the patient at initial presentation. Generalized alveolar bone loss is observed. (d) Axial sections of the computed tomography scan obtained at initial presentation. Diffuse involvement of both maxilla and the mandible is observed.

A 26-year-old male patient presented at the Department of Oral and Maxillofacial Surgery at Akdeniz University with complaints of teeth mobility, intense pain, and difficulty in chewing. His medical history was not significant for any medical conditions. Intraoral examination revealed generalized gingival hyperplasia with a greater severity in the palatal region (Figure 1(a)). The gingival enlargement was sessile, soft in consistency, and red in colour with ulcerated surfaces covered by a necrotic slough (Figure 1(b)). Furthermore, gingival recession, periodontal pockets, bleeding of the oral soft tissues, and halitosis were recorded on clinical examination. Widespread alveolar bone loss consistent with severe teeth mobility was detected on the orthopantomogram (Figure 1(c)). Further radiological examination of the patient with computed tomography scan revealed multiple alveolar lesions with poorly defined and invasive margins (Figure 1(d)).

**Figure 2:**
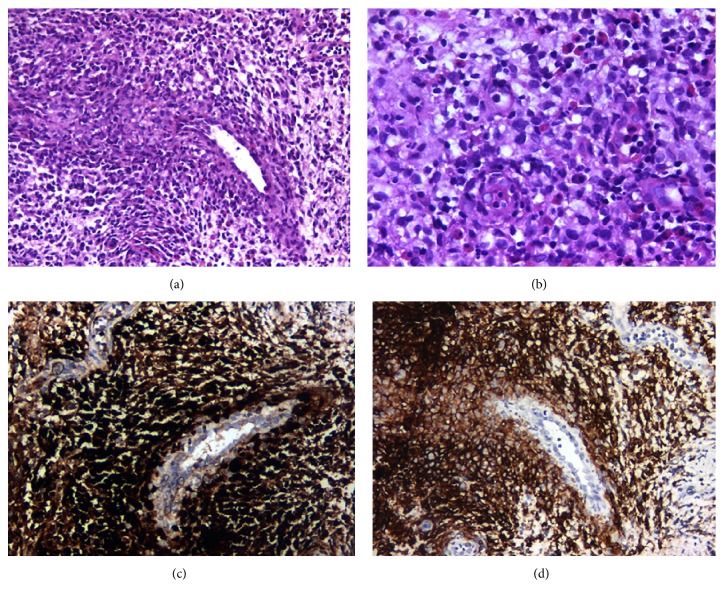
Infiltration of the edematous stroma by polygonal cells with eosinophilic cytoplasm, oval nuclei with longitudinal grooves resembling coffee beans named as Langerhans cells, and eosinophils are observed. (a) Haematoxylin and eosin, ×200 magnification. (b) Haematoxylin and eosin, ×400 magnification. (c) Langerhans cells were highlighted immunohistochemically by S-100, ×200 magnification. (d) Langerhans cells were highlighted immunohistochemically by CD1a, ×200 magnification.

Following extraction of the teeth with severe mobility, periodontal therapy was initiated, focusing on aggressive periodontitis among other differential diagnoses. However, no significant improvement in periodontal conditions of the remaining teeth or the severity of oral lesions was achieved. Consequently, eosinophilic granuloma was strongly considered and an incisional biopsy was obtained from the palate. Histopathological examination revealed bony destruction consisting of Langerhans cells with nuclear grooves accompanied by eosinophils and lymphocytic infiltration (Figures 2(a) and 2(b)). LCs were immunopositive with S-100 and CD1a (Figures 2(c) and 2(d)). On the basis of these findings, the patient was diagnosed with LCH.

**Figure 3:**
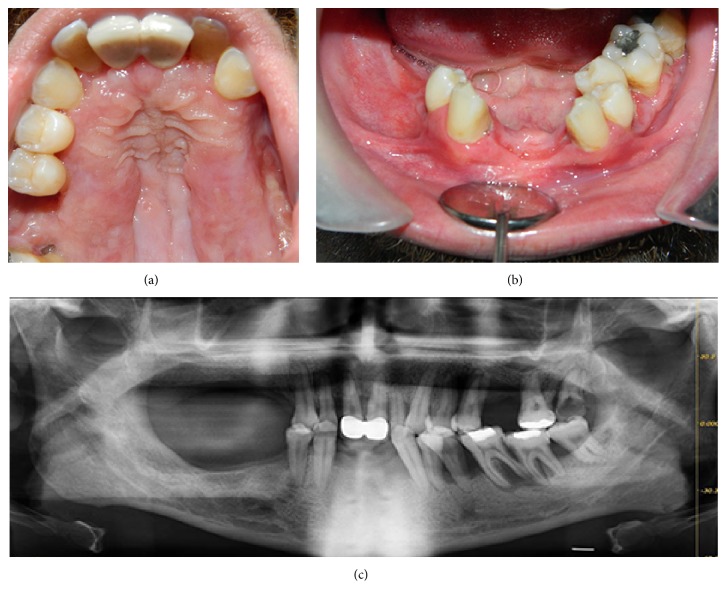
(a) Intraoral view of the patient 12 months after initiation of chemotherapy. Significant regression of gingival lesions is observed on the palatal surface of the maxilla. (b) Intraoral view of the patient 12 months after initiation of chemotherapy. Significant regression of gingival lesions is observed in the mandible. (c) Panoramic radiograph of the patient 12 months after initiation of chemotherapy. Periradicular bone levels of the remaining teeth are maintained except for mandibular left premolar and molar teeth, around which minimal progression of the alveolar bone loss is observed.

The patient was referred to the Department of Hematology for further examination. Radiographic examination of the patient revealed ground-glass lung nodules within the upper lobe on conventional chest radiography, although further radiographic examination with positron emission tomography (PET) and computed tomography (CT) failed to show lung involvement. Blood and coagulation profiles and liver functions as well as urine analysis were found to be noncontributory. A bone marrow biopsy did not reveal any infiltration. On the basis of clinical and radiographical findings, the definitive diagnosis of LCH with unifocal involvement was reached. The patient underwent systemic chemotherapy with vinca alkaloids (Vinblastine 6 mg/m2/week) and corticosteroids (Prednisolone 40 mg/m2/day) and remained under follow-up by the Department of Oral and Maxillofacial Surgery. Regression of gingival lesions, significant decrease in mobility of the remaining teeth, and severity of pain were noted at 12-month follow-up after initiation of chemotherapy (Figures 3(a) and 3(b)). Radiological examination revealed no progression in alveolar bone loss, except for mandibular left premolar and molar teeth, around which periradicular bone loss minimally progressed since the initial presentation (Figure 3(c)). Currently, the patient remains stable and asymptomatic for oral lesions with no further complaints of teeth mobility and pain.

## 3. Discussion

Langerhans cell histiocytosis (LCH) is a rare disorder of the reticuloendothelial system with unknown etiology [12]. It is a clonal disease of myeloid dendritic cells that can affect all age groups but mainly children aged 1–4 years [2]. The diagnosis of LCH is reached by evaluating clinical and radiographic findings and confirmed by histopathological and immunohistochemical studies. Among the histopathological findings, infiltration of Langerhans cells, eosinophilic granulocytes, lymphocytes, and giant cells are regarded as prominent features of the disease. The characteristic immunophenotype of LCH includes expression of CD1a, S100 protein, and langerin (CD207) in LCs. On electron microscopy, elongated, zipperlike cytoplasmic Birbeck granules are observed [1, 13, 14].

LCH most frequently involves the bone and skin, followed by the hematopoietic system, lymph node, liver, spleen, soft tissue, lung, thymus, and pituitary gland [15]. Among the most commonly reported systemic symptoms are development of a soft tissue mass, bone pain, skin rash, fever, and lymphadenopathy [5], while oral manifestations of LCH include hyperplasia of the gingiva or ulcers of the cheek, palate, or tongue mucosa and underlying bone lesions [16].

When encountered in the oral cavity, differential diagnosis of LCH poses a significant challenge for the dental professional, as several clinical features of the disease resemble more common conditions including periodontal disease, malignancies, and granulomatous or ulcerative lesions [4, 17]. Accordingly, clinical and radiological findings of the presented case entailed a primary diagnosis of aggressive periodontitis, which could have indicated near-total extraction. However, reevaluation of the patient and an incisional biopsy for histopathological examination helped authors avoid misdiagnosis of the disease and possibly an incorrect treatment.

Early detection of LCH plays an important role in its prognosis, which is closely related to the age of onset, number of involved organs, and the degree of functional lesion [5]. This is particularly significant when the fact that initial symptoms of LCH may present in the oral cavity is taken into consideration. The authors of the study believe that awareness of oral manifestations of LCH may aid clinicians greatly in reducing morbidity and mortality associated with this debilitating condition. Symptoms that are recognized timely and accurately are of vital importance in reaching a definitive diagnosis and thereby conducting an effective treatment. Early diagnosis and effective treatment of LCH have been documented to not only prevent the progression of disease but also avoid further complications including orthopaedic disabilities, hearing impairment, diabetes insipidus, skin scarring, and neuropsychological defects, chronic pulmonary dysfunction, liver cirrhosis, secondary malignancies such as acute lymphoblastic leukemia or solid tumors, and growth retardation [18–23].

Treatment of LCH is carried out by surgical excision, chemotherapy, radiotherapy, or combination of these modalities [13, 24]. In the present case, the patient had diffuse oral involvement and following the systemic chemotherapy, oral lesions regressed without need of an additional local intervention in the oral cavity.

## 4. Conclusion

The rarity and variable system involvement of LCH necessitate a multidisciplinary approach to be carried out for accurate diagnosis, effective treatment, and an uneventful follow-up. Awareness of oral manifestations of LCH may aid clinicians greatly in reducing morbidity and mortality associated with this debilitating condition.
